# Timing matters: The impact of label synchrony on infant categorisation

**DOI:** 10.1016/j.cognition.2015.02.004

**Published:** 2015-06

**Authors:** Nadja Althaus, Kim Plunkett

**Affiliations:** Department of Experimental Psychology, University of Oxford, United Kingdom

**Keywords:** Categorisation, Cognitive development, Language development, Eye tracking, Visual attention

## Abstract

The impact of labelling on infant visual categorisation has yielded contradictory outcomes. Some findings indicate a beneficial role while others point to interference effects in the presence of labels. The locus of these divergent outcomes is largely unclear. We explore the hypothesis that the timing of the label is of crucial importance, proposing that synchronous presentation of words and objects induces a higher processing load than asynchronous presentation (image onset before labelling). A novelty preference experiment with 12-month-olds reveals that synchronous presentation leads to a diminished preference for a novel object on test in comparison to asynchronous labelling, suggesting a detrimental impact on category learning. However, analyses of infants’ gaze patterns to object *parts* reveal that even synchronous labels do not hinder learning completely. We conclude that synchronous labels interfere with the familiarisation process, but this process involves shifts in familiarity vs. novelty preference rather than overshadowing of visual learning. Besides offering detailed insight into the effects of labelling on infants’ visual attention, these findings offer the potential to reconcile previous contradictory results.

## Highlights

• We report an eye tracking study with 12-month-olds learning a novel category.
• Synchronous labels lead to a decrease in object-level novelty preference.
• Detailed eye-tracking data reveal that this is not due to overshadowing.
• Increased load appears to shift looking towards familiarity preference.
• Our findings reconcile previous contradictory results on the impact of labelling.

## 1 Introduction

Investigations of linguistic influences on cognitive processes have drifted in and out of fashion over the past half century or so. Strong assertions in favour of linguistic determinism (Whorf, 1956) and relativity (Brown & Lenneberg, 1954) have gradually yielded ground to less radical points of view (e.g., Boroditsky, 2001; Hunt & Agnoli, 1991). From a developmental perspective, the investigation of the impact of language on thought is of fundamental importance: do infants use language, and words in particular, as cues to learn about the complex structure of the world? Brown and Lenneberg (1954) were well aware of the developmental implications of Whorf’s thesis:

The world can be structured in many ways, and the language we learn as children directs the formation of our particular structure. Language is not a cloak following the contours of thought. Languages are molds into which infant minds are poured. (Brown & Lenneberg, 1954, p. 454)

The ubiquity of labels in an infant’s environment, both in speech directed at the infant and in conversation between adults overheard by the infant, renders the possibility of linguistic influence highly plausible (Akhtar & Tomasello, 1996). Shared labels can indicate that dissimilar looking things may share attributes or function (e.g., a bonnet and a boater may both simply be called a “hat”). Thus, several studies over the past 20 years have found facilitative effects of labelling on categorisation in pre-linguistic infants between three and twelve months (e.g., Balaban & Waxman, 1997; Ferry, Hespos, & Waxman, 2010; Fulkerson & Waxman, 2007; Waxman & Braun, 2005; Waxman & Markow, 1995).

One focus in this line of research was placed on the question of whether these effects are specific to linguistic labels or can be achieved by any consistent auditory stimulus. In both 6- and 9-month-old infants the facilitation of category formation seems to be restricted to novel labels (Balaban & Waxman, 1997; Ferry et al., 2010; Fulkerson & Waxman, 2007). Three-month-olds appear to benefit equally from non-human primate vocalizations, but not other tone stimuli (Ferry, Hespos, & Waxman, 2013), indicating that infants gradually learn to treat speech as a specific signal.

In addition to the studies demonstrating the facilitation of single-category formation, Plunkett, Hu, and Cohen (2008) have demonstrated that labels serve to guide the formation of category boundaries when the structure of visual space is ambiguous. This work suggests that even infants who are just at the beginning of language development can make use of labels when learning about objects and similarities between them. However, contradictory results which report “auditory overshadowing” effects in the presence of labels, as well as other auditory stimuli (Robinson & Sloutsky, 2004, 2007a, 2007b; Sloutsky & Robinson, 2008), question whether labelling has uniformly beneficial effects for infant visual categorisation. In these studies, labels are considered to have an interfering effect, blocking the formation of object categories. This constellation of findings raises the question as to the conditions under which labels facilitate learning, and what factors may contribute to labels attenuating learning.

From an information-processing perspective, labels undoubtedly provide information that may help learning, e.g., by increasing perceived similarity between objects that share labels (Sloutsky, Lo, & Fisher, 2001), or by highlighting commonalities (Waxman & Markow, 1995). However, processing this additional signal comes at a cost: attention and processing resources have to be allocated to two modalities rather than one. The exact circumstances in which labels are encountered may play a vital role in determining whether they will interfere with, or facilitate, processing. We explore the possibility that the timing of the label is critical: If image and label are presented in exact synchrony, this may impose high perceptual load (Lavie, Lin, Zokaei, & Thoma, 2009; Robinson & Sloutsky, 2007b), and processing in one or both modalities may be attenuated. By contrast, if there is a delay between visual and auditory onset, this may allow infants to process both stimuli equally well because some visual object recognition processes will already have been completed by the time the label occurs (Grossmann, Gliga, Johnson, & Mareschal, 2009; Quinn, Westerlund, & Nelson, 2006).

The question of modality-specific attenuation is particularly interesting in the light of results indicating a transition from auditory dominance in infancy (Lewkowicz, 1988a, 1988b; Robinson & Sloutsky, 2004; Sloutsky & Napolitano, 2003) to visual dominance in adulthood (Colavita, 1974; Posner, Nissen, & Klein, 1976; Sinnett, Spence, & Soto-Faraco, 2007). Studies investigating the developmental trajectory have found visual dominance to emerge between 9 and 10 years of age (Nava & Pavani, 2013), with 4-year-olds exhibiting mixed results (Robinson & Sloutsky, 2004). Robinson and Sloutsky (2004) state two plausible reasons for advantages in auditory processing early in development. The first is that audition develops earlier and is available to the fetus from the third trimester of gestation (Birnholz & Benacerraf, 1983), whereas the visual system only receives external input from birth. This may cause audition to outweigh visually perceived signals early in life. An alternative hypothesis is that audition is initially dominant due to the transient nature of auditory stimuli. According to this argument auditory dominance is directly related to the limited attentional resources available in infancy, which cause attention to be predominantly directed toward the stimulus that needs to be processed immediately. Posner et al. (1976) suggested that visual dominance may emerge in adult sensory processing in order to compensate for the fact that visual signals are less alerting than signals in other modalities. In summary, a hypothesised developmental trajectory is that an increase in attentional resources over development allows the early auditory dominance to disappear, and a visual dominance develops once it becomes advantageous to compensate for the less alerting nature of visual stimuli.

Regarding the processing of object and label pairings we therefore hypothesise that if interference occurs due to the presence of multiple stimuli in the synchronous (but not the asynchronous) condition, visual learning should be attenuated rather than auditory learning.

In addition to the impact of processing capacity there is another argument to be made regarding ecological validity of synchronous vs. asynchronous labelling. Whereas synchronous label onsets have been used in experimental studies reporting interference effects (e.g., Robinson & Sloutsky, 2004, 2007a, 2007b; Sloutsky & Robinson, 2008), asynchronous labelling scenarios are more likely to occur in a young child’s everyday experience, e.g., a caregiver asking “Do you like the ball?” when the child is already attending to the object (Baldwin, 1991). In fact, Tomasello and Farrar (1986) reported that the caregiver’s tendency to name objects already in the infant’s attention (as opposed to re-directing their attention to an object by labelling it) correlated with vocabulary size at 21 months. Similarly, they found an advantage for labelling following the child’s attention in a word learning experiment. Even though some researchers have claimed that synchrony is beneficial to the formation of word-object associations (Gogate, Bahrick, & Watson, 2003), and cross-modal synchrony has been demonstrated to facilitate discrimination of amodal signals such as tempo or rhythm (Bahrick & Lickliter, 2000), it is likely that synchronous picture-word pairings are unusual and surprising to infants at one year of age. These infants, after all, are at a stage in development where they have learned that words often occur together with their referents, but not generally in synchrony like “causal” sounds, such as a hammer hitting a wall. By contrast, recent work using a head-mounted camera demonstrates that word learning is successful in situations where the referent object is brought close to the infant’s face several seconds before the label occurs (Pereira, Smith, & Yu, 2013).

Another potential source of the differential impact of labels is the type of objects used in the respective studies. Investigations reporting a facilitative effect on categorisation often use familiarisation stimuli involving object kinds that the infant may well have encountered before, such as toy animals (e.g., Balaban & Waxman, 1997; Ferry et al., 2010; Fulkerson & Waxman, 2007; Waxman & Braun, 2005; Waxman & Markow, 1995), whereas studies reporting interference effects often involve objects that are entirely novel (see Robinson & Sloutsky, 2004; Sloutsky & Robinson, 2008). No study has reported both interference *and* facilitation effects for the *same set of familiarisation stimuli.* It is therefore possible that the main factor underlying the discrepancy in outcomes is category novelty or complexity. While category complexity may play a role, we will argue in the present paper that the timing of the label is a crucial factor affecting infants looking behaviour. We will examine the impact of synchronous vs. asynchronous presentation of labels with the same set of objects, and demonstrate that synchronous presentation has a deleterious effect on categorisation as compared to asynchronous or silent presentation, in a novelty preference task. Importantly, learning is successful both in silence and with asynchronous labels. Given that the visual stimuli are identical in both cases, category complexity is not a confound in the present case. Previous interpretations of such interference effects invoke “auditory overshadowing” of the visual stimuli during familiarisation (Robinson & Sloutsky, 2004, 2007a, 2007b; Sloutsky & Robinson, 2008). We further investigate this possibility by examining infant attention to object parts during both familiarisation and test. If synchronous labels overshadow the processing of visual stimuli, this should result in information not being encoded. In this case we would expect infants to be impervious to feature distributions of the familiarisation objects. However, if synchronous labels impose a higher perceptual load, visual processing may merely be attenuated without complete failure to encode feature distribution information (Lavie et al., 2009; Robinson & Sloutsky, 2007b). In this case it may still be possible to detect infant sensitivity to the characteristics of the visual object, even in the absence of novelty preference, which is typically used to index category formation.

### 1.1 Overview of study

**Fig. 1:**
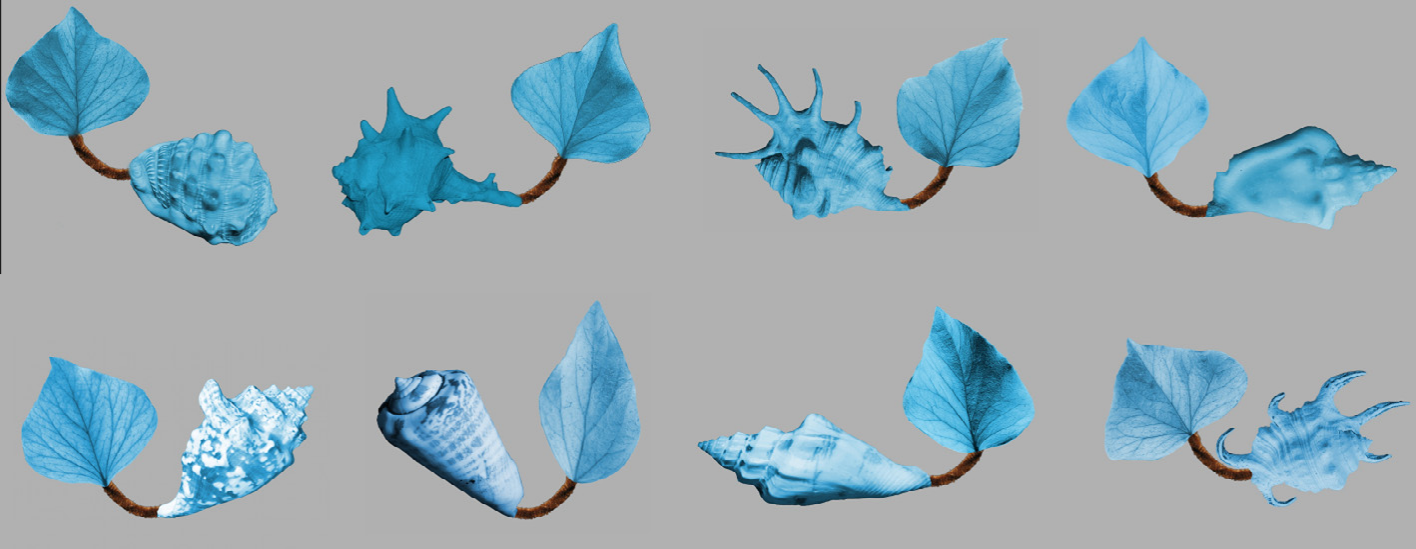
Example familiarisation set.

In order to examine the impact of audio-visual synchrony on infant object categorisation, we familiarised three groups of 12-month-olds with exemplars taken from a novel object category, either in silence (Silent condition), with labels presented one second after the picture onset (Asynchronous Label condition), or with labels and pictures having simultaneous onset (Synchronous Label condition). The stimuli were constructed to contain spatially separate object parts (a shell and a leaf), permitting tracking of infants’ attention at the level of parts, as well as whole objects (Fig. 1). Shell parts were more variable than leaf parts. This enabled us to measure infant sensitivity to object feature distributions, and thereby distinguish between ‘overshadowing’ and ‘perceptual load’ interpretations of any interference effects. The difference in variability also meant that the leaves represented a shared feature, resembling a ‘diagnostic’ part that indicates category membership.

**Fig. 2:**
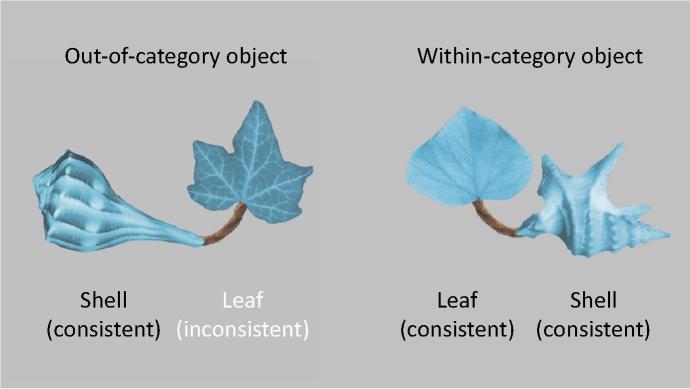
A sample test display illustrating relative novelty of objects and parts.

After familiarisation, infant categorisation was assessed with a novelty preference test trial, in which they were presented with two novel objects side-by-side in silence (see Fig. 2): A within-category novel object contained a leaf and shell that were consistent with the set shown during familiarisation, but had not been shown before. An out-of-category novel object contained a novel but consistent shell and a novel and inconsistent leaf (in other words, the ‘diagnostic’ part was replaced with an inconsistent version). A silent test trial allows probing the boundaries of the category representation formed by the infants, and to compare categorisation across groups, regardless of whether the infants were or were not presented with labels during familiarisation (cf. Balaban & Waxman, 1997; Ferry et al., 2010; Fulkerson & Waxman, 2007; Robinson & Sloutsky, 2007a).

Category formation was indexed by systematic preference for the out-of-category novel object over the within-category novel object (cf. Mareschal & Quinn, 2001; Strauss, 1979).

Examination of object-based novelty preferences in the Asynchronous, Synchronous and Silent conditions permits an evaluation of the importance of the label’s timing in infants’ ability to process and integrate audio-visual stimuli. If category formation is attenuated only in the Synchronous condition, then part-based looking patterns can shed further light on the question of whether this result is due to overshadowing (strong interference) or due to increased perceptual load (weak interference). A lack of systematic preferences for any object part would indicate that synchronous labels overshadow visual processing. By contrast, residual sensitivity to the feature distributions of the separate object parts during familiarisation and/or test would constitute evidence that synchronous labelling imposes a greater perceptual load (without *overshadowing* visual processing).

## 2 Methods

### 2.1 Participants

A total of 87 infants participated in this study (mean age: 372 days, range: 353–386 days, 42 girls). Nine additional infants were not included in the analysis due to failure to reach the looking time criterion (a minimum of 6 familiarisation trials with recorded looking time, and looking time recorded for test trials). Infants were recruited shortly after birth at the local maternity ward and English was the main language spoken in their home.

### 2.2 Stimuli

Candidate members of a novel category were constructed by assembling 9 “objects” from images of a shell, a leaf and a pipe-cleaner (see Fig. 1) in the GNU Image Manipulation Program (GNU Image Manipulation Program, 2013). Across the different objects, the leaves were very similar, the shells highly variable, and the invariable pipe cleaner served as a connecting limb between these two parts. In addition, an “out-of-category” object was constructed (see Fig. 2) to contain a shell that was consistent with the category, but an inconsistent type of leaf. All images were depicted against a medium grey background. Objects subtended approximately 14° × 10° visual angle. On the test display, there was a gap of approximately 5° visual angle between out-of-category and within-category objects. The location of the out-of-category object (left or right) was counterbalanced across subjects. Previous eye tracking research has shown that subjects are more likely to direct a fixation at a point near the centre of the screen than further away (Buswell, 1935; Tseng, Carmi, Cameron, Munoz, & Itti, 2009). To prevent this centre bias from confounding looking preferences in the test trial, the two objects were always oriented in such a way that the inconsistent leaf in the out-of-category object as well as the consistent leaf in the within-category object were close to the centre of the screen (see Fig. 2). This permitted direct comparison of looking directed at these two parts. As position and orientation of the familiarisation exemplars were counterbalanced, both test stimulus positions/orientations were equally familiar to the infants at test. A recording of the novel label “timbo”, pronounced by a female British–English speaker in an infant-directed voice, served as the auditory stimulus.

### 2.3 Procedure

After a short warm-up phase during which written consent was obtained from the caregiver, infants were seated on the caregiver’s lap at 75 cm distance from the eye tracker. A nine-point calibration sequence was performed up to three times or until all points had been calibrated successfully.

One third of the infants (N=29) were allocated to the Silent, Asynchronous Label and Synchronous Label conditions, respectively. Infants were presented with eight out of the nine familiarisation objects in pseudo-randomised order, each for 6000 ms. The remaining object from the familiarisation category served as the within-category object on the test trial. Four of the familiarisation objects appeared on the left half of the screen, and four on the right, in no predictable order. Every image was preceded by an attention getter, a small animation at the centre of the screen (with a medium grey background) accompanied by an attractive chiming sound. Animation and sound lasted about 1500 ms, with the next trial beginning 2000 ms after the onset of the attention getter. In the Asynchronous Label condition, the sound file containing the label “timbo” (duration: 800 ms) was played 1000 ms after picture onset. In the Synchronous Label condition, the label started at picture onset. Familiarisation was followed by the test trial, which lasted 10,000 ms. On the test trial, the test object described above was paired with the remaining object from the familiarisation set. The test trial was conducted in silence. Infants’ looking was recorded using a Tobii eye tracker sampling at 120 Hz throughout the familiarisation and test phase.

## 3 Results

We first report global measures of looking during familiarisation (i.e., with respect to whole objects), and then turn to a more detailed analysis of looking directed at individual object parts. Areas-of-interest (AOIs) were defined to contain the area covered by the images of shell and leaf, respectively, plus a 30-pixel margin around the image outline (corresponding roughly to the eye tracker’s 0.5° visual angle accuracy). Recorded gaze data were analysed using custom Matlab code.

### 3.1 Looking time during familiarisation

**Fig. 3:**
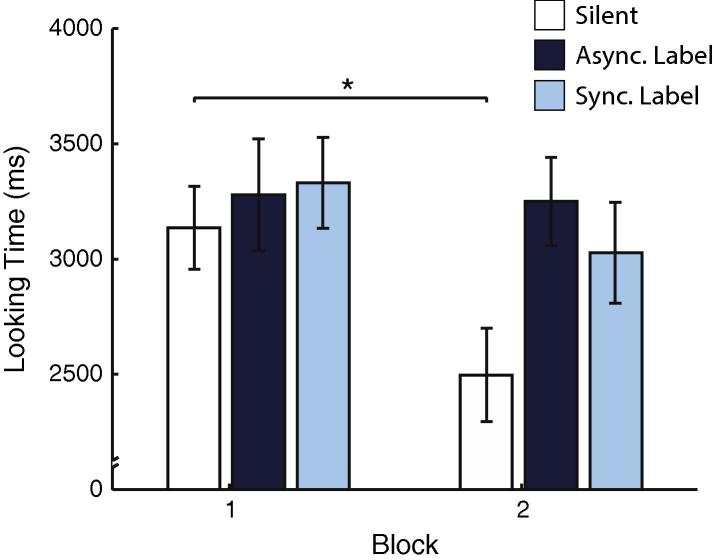
Looking time during familiarisation.

Looking time for each familiarisation trial was calculated as the sum of fixation time falling on the leaf and shell AOIs. In order to assess whether infants had begun to habituate by the end of the familiarisation phase (a typical indicator of learning), we divided the eight trials into two blocks of four trials (e.g. Eimas & Quinn, 1994). Average looking times for Blocks 1 (Trials 1–4) and 2 (Trials 5–8) are shown in Fig. 3. The data were submitted to a mixed model ANOVA with within-subjects factor Block (Block 1, Block 2) and between-subjects factor Condition (Silent, Asynchronous, Synchronous). This yielded a significant main effect of Block (*F*(1, 84) = 6.464, *p* = .013). While the Block × Condition remained non-significant (*F* = 1.870, *p* = .161), as did the main effect of Condition (*F*(2, 84) = 1.99, *p* = .143), planned comparisons showed that infants’ looking in the Silent condition did decrease (*t*(28) = 3.575, *p* = .001). In the two conditions with labels infants’ attention did not appear to decrease (Asynchronous: *t*(28) = .112, *p* > .91; Synchronous: *t*(28) = 1.42, *p* > .17; all paired, 2-tailed tests). These findings are consistent with previous research showing that auditory input maintains infant looking (Baldwin & Markman, 1989; Plunkett et al., 2008; Robinson & Sloutsky, 2007a) during a sequence of familiarisation presentations.

### 3.2 Part-based looking during familiarisation

To investigate whether synchronous or asynchronous labels affected infants’ processing of individual parts during familiarisation, we calculated a mean looking proportion for the “leaf” part by dividing the amount of looking at the leaves by the amount of looking at both object parts (leaves and shells) for each 6-s trial and obtaining the average across the familiarisation phase. A one-way ANOVA showed that the proportion of time that infants spent looking at leaves did not differ across conditions (*F*(2, 84) = .64, *p* = .53). Overall, they spent less time looking at the leaves than at the shells, indicating that they were sensitive to the greater variability of the shells in all conditions. (Proportion of looking at leaf, collapsed across conditions: *M* = .35, *SE* = .11; *t*(86) = 12.9, *p* < .0001.)^1^ This finding suggests that synchronous label presentation does not overshadow visual processing during familiarisation.

### 3.3 Object-based novelty preference at test

**Fig. 4:**
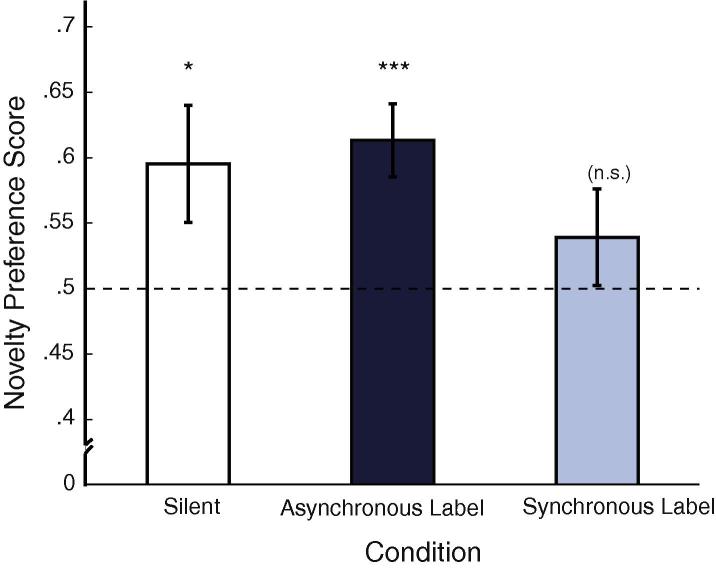
Novelty preference scores on test: ^∗^ indicates a result significant at the .05-level, ^∗∗∗^ indicates a result significant at the .001-level.

Object-based novelty preference scores at test were obtained by dividing the amount of looking time at the out-of-category object by the total looking time accumulated for the trial (within-category and out-of-category objects). Novelty preference scores were normally distributed in all conditions (Shapiro–Wilk, all *p*s > .65). The results are given in Fig. 4. A one-way ANOVA did not reveal differences between the conditions (*F*(2, 86) = 1.08, *p* > .34). Importantly, however, we also conducted planned comparisons against chance for each condition separately. If infants failed to form a category and did not discriminate between the two novel objects, we would expect them to spend approximately 50% of their looking directed at each object. By contrast, if they successfully formed a category, we would expect them to reliably prefer the out-of-category over the within-category novel object. Therefore, planned comparisons against chance (0.5) were conducted for each condition. Infants demonstrated systematic novelty preferences in the Silent and Asynchronous Label conditions (Silent: *t*(28) = 2.13, *p* = .04; Asynchronous: *t*(28) = 4.037, *p* < 0.001) but not in the Synchronous Label condition (*t*(28) = 1.066, *p* = .295, all two-tailed one-sample *t*-tests against chance).

We also identified the number of infants in each condition who spent more than 50% of looking time at the novel, out-of-category object. This analysis confirmed that a significant number of infants in the Asynchronous Label condition demonstrated a novelty preference (*N* = 22, total: 29, *p* < .01). For the Silent condition there was a trend in the same direction (*N* = 19, total: 29, *p* = .13), but the number of infants demonstrating a novelty preference in the Synchronous condition did not differ from chance (*N* = 16, total: 29, *p* > .7, all two-tailed binomial tests). These findings suggest that infants formed a category during familiarisation in the Silent and Asynchronous Label conditions, but not in the Synchronous Label condition.

### 3.4 Part-based looking at test

Infants’ failure to recognise the out-of-category stimulus as novel in the Synchronous condition suggests a detrimental impact of the synchronous label on category learning. Yet, the analysis of part-based looking during familiarisation revealed that infants were sensitive to the greater variability of the shell in all 3 conditions. We now examine whether this part-based sensitivity extends to the novelty preference test.

**Fig. 5:**
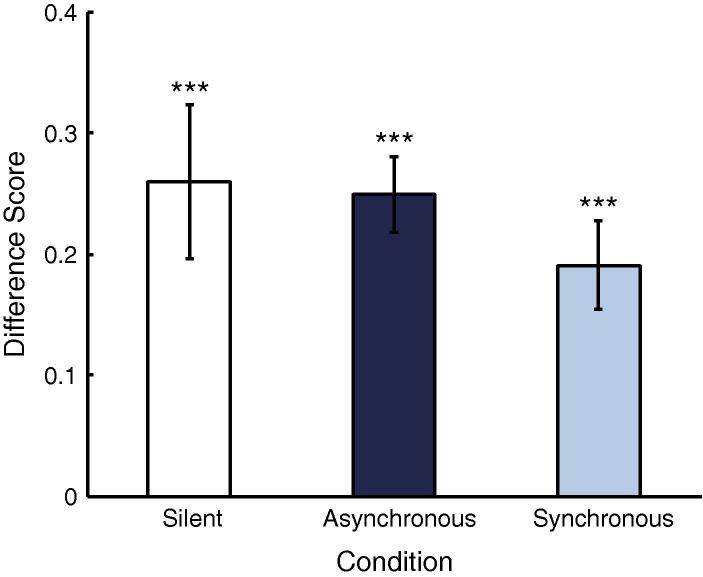
Difference scores (proportion out-of-category leaf – proportion within-category leaf) for all conditions. ^∗∗∗^ Indicates a result significantly above 0 at the .001-level.

To this end we calculated a difference score for the looking proportions directed at the two leaves (i.e. proportion of looking directed at the out-of-category leaf minus proportion of looking directed at the within-category leaf, out of looking directed at any part) across the 10-s test trial for each infant (see Fig. 2 for a sample test display). A positive difference score indicates more looking at the out-of-category leaf. Difference scores were normally distributed in all conditions (Shapiro–Wilk, all *p*s > .38). Fig. 5 shows the difference scores for all three conditions. A one-way ANOVA was far from significant (*F*(2, 86) = .65, *p* > .522). In all three conditions, infants’ difference scores were clearly larger than zero (after collapsing the conditions: *M* = .23, *SE* = .03). In particular, infants preferred the novel leaf in the Synchronous condition (*M* = .19, *SE* = .04; 2-tailed one-sample *t*-test: *t*(28) = 5.17, *p* < .001). In contrast to the global looking measure, the part-based measure shows that infants in the Synchronous condition did not fail to encode the distributional properties of the leaf, as they perceived the novel leaf as unfamiliar.

Dividing the infants in the Synchronous condition into two groups according to their object-level performance clarifies the results further (see Table 1). Infants with more than 50% looking at the out-of-category object (*N* = 16) achieved an average difference score of 0.28 (*t*(15) = 5.15, *p* < .001, one-sample *t*-test against zero, two-tailed). In this respect, their looking is similar to infants in the Asynchronous condition. However, even the superficially unsuccessful infants (*N* = 13) who spent less than 50% of the trial looking time on the out-of-category object, obtained an average difference score of .09 (*t*(12) = 2.66, *p* = .02). Clearly, even those infants with an overall preference for the familiar object responded to the novel leaf.

## 4 Discussion

We familiarised infants with a novel object category either in silence (Silent condition) or with novel labels, which were either presented one second after image onset (Asynchronous condition) or simultaneously with image onset (Synchronous condition). On a subsequent novelty preference test trial, infants in both the Silent and the Asynchronous condition showed a systematic preference for the out-of-category object, indicating that they had successfully learned the target category. Infants in the Synchronous condition, by contrast, did not exhibit a systematic preference. Using the established measure of object-based novelty preference as a marker of successful category formation this would be interpreted as a failure to learn the target category. However, a more fine-grained analysis of part-based looking suggests otherwise. Infants in the Synchronous condition, even those who exhibited below-chance object-based novelty preference, looked longer at the novel leaf (out-of-category object) than at the corresponding part in the within-category object. Furthermore, infants looked longer at the more variable shell part during familiarisation, irrespective of condition. These clear responses to novelty demonstrate that they did not fail to learn in the Synchronous condition.

Our results imply, first of all, that timing matters: infants’ object-based performance was affected by synchronous label presentation. Differences between synchronous and asynchronous label presentation offer important cues as to how words and images are processed. Since the onset timing of the auditory relative to the visual stimulus affected infants’ behaviour, it is clear that stimuli are processed on-line at the time the two components are perceived, rather than stored in short-term memory and processed separately and independently of their presentation time. In the latter case, timing should not matter, so such a delayed processing strategy can be excluded.

What is the nature of the processes that are initiated upon the infant’s perception of a visual object, and an auditory label, respectively? Firstly, whereas the visual image tends to be available for potentially long periods of time, auditory information is fleeting. This difference has been used as a potential explanation for auditory dominance in infancy (Robinson & Sloutsky, 2004), where limited attentional resources may restrict detailed processing to one sensory domain. Secondly, information transmission is inherently different in the two domains. In terms of information becoming available to the sensory systems, vision is instantaneous (i.e. the whole image is available simultaneously), whereas auditory information unfolds over time. For speech perception in particular this implies a considerable lag between perception of the onset sound, and the time at which a word can be identified unambiguously. As the incoming speech signal is processed, a cohort of lexical candidates with matching onset is activated (Marslen-Wilson & Welsh, 1978) and deactivated as more information becomes available. In adults, this gives rise to a cascade of spreading activation in the lexical system, with a cohort of phonologically compatible lexical matches being activated before semantic matches (Huettig & McQueen, 2007). Mani and Plunkett (2011) demonstrated that similar cohort effects can be observed in toddlers less than a second after word onset. It therefore seems plausible that a precursor of the cascading activation, which in adult lexical processing results in phonological and semantic priming, is triggered even in 12-month-olds by hearing a word.

Similarly, observing a visually presented object will trigger a cascade starting with early visual processes and eventually resulting in a category-level representation. Electrophysiological studies have indicated that category assignment in infants occurs as early as 300–500 ms after stimulus onset (Grossmann et al., 2009), rendering the first second of exposure a crucial time interval for visual processing. Mani and Plunkett (2010) have further provided evidence for implicit naming in young infants – in other words, these early visual processes will eventually give rise to a phonetic representation.

In the present study, timing differences affected infants’ processing of visual images even though every picture was on the screen for several seconds after the label had occurred. This indicates that processes occurring at the *beginning* of exposure to a visual stimulus are crucial for learning. We can only speculate at this stage whether it is this cascading activation process (leading from visual to semantic activation) that is disrupted in the presence of synchronous labels, which triggers its own cascade leading from auditory to semantic activation, but spared with a one-second delay. If hearing words triggers a whole cascade of (pre-)lexical processes that may go on well beyond the physical duration of the auditory signal, this could further prevent infants from “catching up” with processing in the visual domain once the auditory signal has passed.

The results from the present study indicate clearly that infants’ response to the target stimuli differs on the test trial. Whether the lack of novelty preference observed under synchronous labelling reflects differences in infants’ mental representation of the target category remains an open question.

However, it is also evident from the results that the impact of synchronous labels is not so detrimental as to disrupt infants’ learning entirely, as shown by the part-based novelty preference. Infants were clearly sensitive to the novel part. The *strong* overshadowing hypothesis, involving a deficit in the encoding of the visual stimuli that would prevent infants from responding to novelty, therefore does not appear to be supported.

One possibility is that the discrepancy between Synchronous and Asynchronous conditions reflects an altered course of habituation processes, e.g. a delay in the shift from familiarity to novelty preference (Hunter & Ames, 1988), as suggested by our “weak hypothesis”. Infants who heard synchronous labels were perhaps just at the threshold to novelty preference as the test trial occurred. In fact, the variability of individual preference scores suggests that infants were at different stages in this process. This is in line with the hypothesis that synchronous labels increase processing load, but rather than involving overshadowing effects that interfere with the encoding of visual stimuli, it is infants’ on-line looking behaviour that is changed.

This distinction is crucial for any account of how infants integrate words and objects over the course of development. The difference between synchronous and asynchronous presentation highlights the fact that word and object processing at this early stage in development are still fragile. At the same time our results also indicate that the difficulties arising due to simultaneous presentation with two complex, as well as novel, stimuli can be overcome. Clearly some visual learning took place, even though the lack of object-level novelty preference indicated a slower, or less complete, learning process. Robinson and Sloutsky (2007b) reported a delayed novelty preference in a continuous habituation paradigm when visual stimuli were accompanied by an unfamiliar novel sound, but this effect was attenuated after pre-familiarisation with the novel sound. Our results are consistent with this notion of decreased processing speed in the presence of unfamiliar auditory stimuli. It seems plausible to hypothesise that even infants who heard labels in synchrony with the visual onset should be able to achieve novelty preference given more exposure to the stimuli.

These findings also inform the earlier-mentioned issues relating to the lack of ecological validity of synchronous audio-visual presentation. As discussed previously, whereas object-then-label scenarios have been shown to be highly effective for word learning (Pereira et al., 2013; Tomasello & Farrar, 1986), synchronous object-label occurrences are unlikely to occur in the infant’s natural environment. The reason for this is simply that the appearance of an object is not causally linked to the occurrence of a label. This raises the question as to whether the “deficit” in processing observed is due to the inherent properties of synchronous presentation, or whether it could be argued that it is merely the unusualness of encountering stimuli in such a way that is problematic. We believe the former to be the case. On the one hand, assuming synchronous stimuli were *not* unusual, one might expect infants to develop mechanisms of dealing with synchrony. An “unusual” stimulus timing, by contrast, would not necessarily be predicted to cause problems unless it were accompanied by a change in cognitive load. Another scenario for object-label pairings would be an asynchronous presentation with the label occurring *before*, rather than after, the visual onset (e.g. Parise & Csibra, 2012). This is something infants experience whenever a caregiver attempts to verbally direct the child’s attention to an object they are not yet attending to. Based on the hypothesis that sequential processing of auditory and visual stimulus (regardless of order) should incur less load than synchronous processing, one would predict that label-then-object presentation should result in equally good performance as object-then-label presentation. However, Tomasello and Farrar (1986) reported that the proportion of such directive utterances made by mothers during an observational study was negatively correlated with vocabulary size at 21 months. One possibility is that label-then-object presentations involve greater memory load, as the label is a transient stimulus and has to be remembered when the object appears, whereas in the object-then-label scenario the object remains visible during and after the presentation of the label. Whether an object-then-label scenario is indeed the optimal scenario for 12-month-olds’ processing of words and objects, or whether labelling prior to object appearance is equally effective, is therefore ultimately subject to further empirical work.

In contrast to previous findings, we do not find strong evidence for a facilitation of categorisation in the presence of asynchronous labels compared to learning in silence. Apart from an increase in the number of successful infants (see Table 1), both groups exhibited similar levels of novelty preference. That 12-month-olds will successfully form a category over a set of objects in silence is not surprising *per se* – the discrepancy with other findings such as those reported by Waxman and colleagues simply indicates that perhaps the stimuli in other studies were more complex or difficult to categorise for infants. One possibility is that our stimuli, being rich photographic images, are more engaging than the line drawings used by Fulkerson and Waxman (2007) and others, and at the same time the category structure is less complex (comparable to a basic, rather than a superordinate level category) than those used by Waxman and Markow (1995). A question subject to further research is whether infants’ mental representation of the target category is identical or different in the silent and asynchronous label scenarios. While both groups exhibit similar levels of novelty preference at test, previous research suggests that labels direct attention to commonalities (Althaus & Mareschal, 2014). It is therefore unlikely that infants ignored the labels in the asynchronous condition. While labels have not improved the already high novelty preference scores beyond those achieved in the silent condition, it seems plausible that their mental category representation has been altered by hearing labels.

Our findings demonstrating reduced novelty preference under synchronous labelling offer the potential to reconcile previous contradictory findings regarding the impact of labelling on categorisation. Studies which report facilitatory effects of labels (e.g., Ferry et al., 2010; Fulkerson & Waxman, 2007; Plunkett et al., 2008) used a delayed label onset similar to our Asynchronous Label condition, whereas Robinson and Sloutsky (2004, 2007a, 2007b) and Sloutsky and Robinson (2008), who report auditory overshadowing, presented auditory stimuli at picture onset. Of course, it is important to acknowledge that label synchrony need not be the *only* factor that determines the impact of labels on infant visual categorisation. The processing load imposed by any coupling of visual and auditory stimuli is also dependent on the visual and auditory complexity or novelty. Robinson and Sloutsky (2007b) found, as mentioned above, that infants’ novelty preference is delayed when familiarisation is accompanied by an unfamiliar sound, but this effect is attenuated when infants are pre-familiarised with this novel sound. Similar mechanisms may apply in the visual domain. The role of category complexity remains elusive, as categories of varying complexity have been employed in past research on the impact of labelling on categorisation but without systematic comparison. Further research will be necessary in order to determine whether the timing of the auditory stimulus can indeed explain the discrepancies between the findings. However, the current research demonstrates that label synchrony is an important determinant of object preferences in novelty preference tasks.

The discrepancy between global looking preferences and part-based results further highlights the limitations of preferential looking as a measure of learning. A decrease in novelty preference scores at the object level does not necessarily imply disruption of visual learning, but can potentially be explained by changes in the speed in which the shift from familiarity to novelty preference is obtained (Hunter & Ames, 1988). Null preferences therefore have to be interpreted with caution, specifically when comparing conditions that inherently differ in terms of processing load, such as a silent condition vs. one that includes auditory stimuli. The discrepancy we report between *object-based* and *part-based* looking illustrates how more sensitive measures may be obtained with careful stimulus design.

In the context of cross-modal processing, synchrony is often claimed to be beneficial, at least for young infants (Gogate et al., 2003). We have argued that the increased processing load due to synchronous presentation appears to slow down category learning. In addition, infants’ cross-modal experience with objects and labels would also appear of central importance. In natural settings, the likelihood of a label occurring at exactly the same time that an object comes into view is rather small. In fact, Pereira et al. (2013) report higher word learning success in toddlers for scenarios in which the labelled object is brought close to the child’s face several seconds prior to the naming event. In terms of learning, asynchronous presentation may offer computational advantages. The opportunity to process visual and auditory information sequentially could be a facilitating factor in the extraction of more complex visual structures – and specifically the kind of abstract similarity needed for categorisation. To paraphrase Brown and Lenneberg (1954), asynchronous language provides moulds into which infant minds are poured.

## Figures and Tables

**Table 1:** Difference scores for leaf looking split by novelty preference scores (NP), for all conditions.

Condition	Infants with NP > .5		Infants with NP < .5	
	*N*	Difference score	*N*	Difference score
Silent	19/29	.38∗∗∗	10/29	.02
Asynchronous	22/29	.30∗∗∗	7/29	.08
Synchronous	16/29	.28∗∗∗	13/29	.09 ∗

∗ A result significantly different from 0 at the .05 level.

∗∗∗ A result significantly different from 0 at the .001 level.

## Footnotes

1 Note that infants did not appear to have an *inherent* preference for the shell. Proportion of leaf looking during the first familiarisation trial, which would reflect any a priori preferences, did not differ from chance in any of the conditions (all *t*s < 1.63, *p*s > .11, all two-tailed *t*-tests).
